# Mothers and Children Exposed to Intimate Partner Violence: A Review of Treatment Interventions

**DOI:** 10.3390/ijerph15091955

**Published:** 2018-09-07

**Authors:** Kimberley Anderson, Elisa van Ee

**Affiliations:** 1 Psychotraumacentrum Zuid Nederland, Reinier van Arkel Groep, 5211 LJ ’s-Hertogenbosch, The Netherlands; e.van.ee@reiniervanarkel.nl; 2 Department for Medical Psychology & Medical Sociology, University of Leipzig, 04103 Leipzig, Germany; 3 Behavioural Science Institute, Radboud University, 6500 HE Nijmegen, The Netherlands

**Keywords:** mother-child, intimate partner violence, family violence, parenting, treatment, intervention

## Abstract

Although a growing field, much is still unknown about how different clinical and social care services might improve outcomes for female victims of intimate partner violence (IPV) and their children who are indirectly exposed to it. This review sought to characterize the structure of programs that have been tested and documented in existing literature, and the mechanisms by which change, if any, may occur. Seventeen individual interventions and two follow-ups (*n* = 19) were included in the review. Findings suggest that a multileveled program of mothers and children working both separately and jointly together across sessions might generate the most successful psychosocial recovery for mothers and children who have experienced violence in the home. The mechanism by which this happens is likely a collaborative one, focused on enhancing the dyadic interaction. This article adds to the growing evidence base on IPV and confirms the positive impact on well-being that programs for IPV victims can have. The evidence-base overall could benefit from testing and replicating a combination of the results found in this review.

## 1. Introduction

Intimate partner violence (IPV) is defined by the World Health Organization [1] as “behavior by an intimate partner or ex-partner that causes physical, sexual, or psychological harm, including physical aggression, sexual coercion, psychological abuse or controlling behaviors” (p. 1). An important addition to this definition is that IPV can occur “between those aged 16 or over who are or have been intimate partners or family members, regardless of gender or sexuality” [2] (p. 1). Despite the acceptance that IPV occurs regardless of gender, women are still predominantly targeted, with estimates varying greatly across the world. One study of particular significance reached a sample of over 24,000 women in 15 different countries. The authors of this study documented the lifetime prevalence of physical or sexual violence as ranging vastly, from 15% in Japan to 71% in Ethiopia [3]. There is comparatively less knowledge regarding the number of men who experience IPV, ranging from: 11.5% in a study of more than 4600 in Sweden [4]; 15.9% in a sample of over 15,000 in the United States [5]; to 22% in a sample of 1700 in Hong Kong [6]. This disparity may be a result of many factors including cultural norms, the quality of reporting processes, defined terminology or underreporting of such crimes, but it underlines the still ever-present risk for women to experience violence in intimate relationships. It is this risk of a woman’s potential exposure to IPV and her typical role as primary caregiver, that means it is highly likely her experiences may indirectly extend to her children, who become *secondary victims* (we use the term secondary victims in this article to highlight the real situation where children are not the direct receiver of violence but experience its effects indirectly (see also Jaffe, Crooks and Wolfe [7]). The mother in this example is the primary victim of violence under the circumstances of IPV, and her child is experiencing the impact of IPV on their mother and thus in her parenting).

For children who do not experience violence directly in the home, the distinction between witnessing IPV and being exposed to it is an important one. Exposure is used more recently to encapsulate the idea that children are known to experience IPV through their awareness of violence between their parents, even if they do not (always) directly witness any violent acts [8,9]. The number of adults who report having been exposed to IPV during childhood can range anywhere from 8% to 25% [10]. In the United Kingdom, approximately 1 million children report having been exposed to IPV [11], but of course, children do still witness violence in the home. In the United States around 80% of children living in violent homes personally observed IPV towards their mother [12,13].

The effects of IPV on mothers and children can manifest in multiple ways. Very often, mothers and children do not acknowledge or discuss violence in the home once it ends [14], and exposure to it can impact the individual functioning of both the mother and the child, as well as affecting their dyadic relationship. For women who directly experience IPV, outcomes are well-documented. As well as physical injury, Graham-Bermann and Miller [15] highlight the increased risk of long-term health concerns such as asthma, stroke and heart disease that impact women who experience violence. Psychologically, women are also at great risk of predominantly posttraumatic stress disorder (PTSD), anxiety and depression, but also suicidal behavior, sleep and eating disorders, social dysfunction, and an increased likelihood of substance abuse [16]. With regard to children, some may personally observe violent acts in the family home, or witness controlling or coercive behavior towards their mother [17]. In situations of violence, research has shown that children as young as one can display heightened distress in response to even verbal conflict between parents [18]. Moreover, witnessing severe IPV has been associated with trauma symptoms, behavioral problems, as well as increased risk of alcoholism, illicit drug use and depressed affect in later life. It has also be linked in one study to perpetration of violence, as a result of social learning [19]. Exposure to violence in the home may also impact the likelihood of adverse psychosocial outcomes [9].This can include: poor emotion regulation, anxiety, depression, low self-esteem, attention difficulties, disturbances in interpersonal relationships and reduced overall adaptive functioning, [12,20,21,22], as well as maladaptive cognitions regarding the causes of IPV; i.e., blaming the mother or themselves [13]. As a result, children can be observed to have reduced problem solving abilities later on within both interpersonal and environmental situations [12].

As well as witnessing violence, growing up with a mother who is impacted by violence herself can reflect in the behavioral functioning of a child [23]. Symptoms of depression observed in women experiencing IPV have been linked to a poorer overall quality of parenting, which in turn, is believed to increase the likelihood of distress and internalizing behaviors in children [24]. Furthermore, Jouriles et al. [20] note a lack of parental warmth and affection as associated with greater dysfunctional behavior in children exposed to violence in the home. Moreover, mothers may also be more punitive with their children, an act that is further linked to internalizing and externalizing displays of child behavior, with high co-occurrence of both types of problems in children who are more regularly and harshly punished [25]. One reason mothers may employ such parenting strategies is to ensure their children are well-behaved, thus avoiding aggravating the abuser [14]. Though the experience of IPV on the dyadic mother-child relationship often results in poor functioning, this is not always the case. In times of stress, parents can act as ‘emotional anchors’, and demonstrate adaptive coping mechanisms to ensure their child’s optimal well-being [13], thus buffering against poor socio-emotional outcomes. Mothers affected by IPV have been observed to be more responsive and warm towards their children, which may act as a protective factor against the negative impact of being exposed to IPV in the family home [26], and play a key role in mediating the distress of chaotic family situations [27].

As a result of the range of outcomes for mothers and children affected by IPV, treatment and support that addresses their individual needs can be successful in targeting their functioning and improving outcomes. Equally, improvements in this domain may have the added benefit of also improving the relationship overall. Interventions that focus on children commonly aim to address the most severe outcomes (e.g., attachment disorders, PTSD, anxiety and conduct problems), usually taking place in shelters or in community-based centers [28]. In addition, McWhirter [29] stresses the importance of allowing space for children to gain understanding and perspective about the event, appraise the safety issues involved, identify and learn to approach the safe people in their life, and master confidence in themselves and their environment. Other important goals for child interventions have been to enhance coping skills; improve communication skills; explore conflict resolution and problem-solving skills; expressing feelings; and changing maladaptive behavior [30]. Age is important for children as secondary witnesses. In households with IPV, younger children are the most likely to witness violent acts [31], yet they have also shown to be more receptive to their mothers’ improved well-being after receiving treatment, compared with older children. One study found youth and adolescent children (aged 6–18 years) as having the least improvement on internalizing behaviors following treatment of their mothers, with many remaining within clinical range after two years. This was compared to children between ages 18 months and 5 years who were more receptive to their mothers’ treatment [32]. Interventions that encompass a wide age-range actually inhibit the ability to identify effects or processes more unique to a particular developmental period [31]. Often interventions for improving mothers’ functioning mirror those for their children (e.g., enhancing problem-solving and communication, as well as how to express one’s feeling), but they also include aspects of developing parenting skills and decreasing parenting stress, how to develop safety plans for their family and how to connect with the community [30].

While interventions of individual sessions allow the space for mothers and children to explore their own issues and receive age-appropriate support, time spent together in joint sessions is believed to help sustain any positive changes within the family unit. A combination of both likely brings about the most long-lasting impact on relationships and well-being [13]. However, research over the past two decades has tended to explain mother-child relationships by exclusively examining a mother’s parenting.

Given the range of evidence on the interaction of individual experiences of IPV (directly as a mother, and indirectly as a child) and the subsequent impact on behavior and functioning–as well as the impact of these on relationships–this review has a primary focus of distinguishing how different types of interventions in psychosocial care settings adapt to meet the needs of mothers and children, both separately and in joint sessions. We aim to do this by (1) mapping existing interventions for women who are directly affected by IPV, and their children who are secondary victims; and (2) to build on similar existing work [28,30,33] by further exploring how the structure and content of intervention programs can bring about change (be it improvements in individual functioning or in dyadic relationships). We strongly feel that understanding the mechanisms underpinning the interventions themselves is useful for guiding future research/clinical practice that seeks to support mother-child dyads affected by IPV.

## 2. Materials and Methods

### 2.1. Eligibility Criteria

This review included interventions that address IPV within families, with children up to 18 years, with any outcome measure, from any country and written in English. This initial wide scope was to ensure all efforts to address the research objective were captured in the literature search and not restricted by article indexing. This review excluded: (1) male victims and programs directed specifically toward fathers and their children to concentrate on mothers only; (2) case studies, as it is not possible to compare results due to a lack of control data; (3) mothers with substance misuse problems as it is not possible to pre-determine the role of these in situations of IPV, and would require an additional level of treatment; and (4) children as direct victims of violence in the home, the reason for this being that although the experiences of children who are exposed to IPV overlaps somewhat with that of child abuse victims–experiencing neglect, abandonment, threats, and acts of abuse [34]–excluding victims of child abuse allows us to study interventions that focus on the effects of the relational trauma of IPV.

### 2.2. Search Strategy

To ensure a comprehensive search of existing literature was conducted, the electronic databases MEDLINE, psycINFO, PILOTS and the Cochrane Library were screened, and publication bias was minimized by including conference papers and book chapters, searching grey literature, and corresponding with authors to identify additional or unpublished work where necessary. The search strategy was defined as terms containing adjectives or derivatives of *mother*, *child*, *intervention* and *intimate partner violence* that were then combined using a series of Boolean AND/OR operators. Derivations of the search terms were combined and adapted to each database accordingly. The combination of terms was deliberately broad to increase sensitivity of the search and identify all interventions. Reference lists of key studies and identified reviews were searched and relevant papers obtained (backward snowballing), as well as finding citations to studies documented (forward snowballing). Initial searches and data extraction were completed on 31 June 2017. Eligible full-text articles were discussed, and consensus was reached on the final inclusion of studies. Further details are available to view in Appendix A.

### 2.3. Data Analyses

A three-stage process for reviewing search outcomes was conducted. First, based on the inclusion criteria, all search results were assessed by title and abstract. Second, if these criteria were met (or if the review of title and abstract was insufficient to exclude the study) the article was retrieved for full review. After full review, a final number of articles were included in the study. Full text articles were analyzed according to intervention population, program structure and treatment mechanisms, as well as outcomes and overall effectiveness. Quality of evidence was guided by the GRADE (Grades of Recommendation, Assessment, Development and Evaluation) approach [35,36,37], which is a systematic and transparent method to reflect the confidence that a guideline adequately supports the effect of a particular treatment recommendation. The GRADE method judges the quality of evidence and strength of recommendations regarding clinical outcomes that matter to clients and reduces bias in interpretation of results by using a standardized scoring system. Randomized Controlled Trials (RCT) start as high quality (4), and observational studies start as low quality (1), Studies are then appraised for risk of bias (limitations of detailed design and execution), heterogeneity, applicability and quality of reporting.

## 3. Results

### 3.1. Search Results

**Figure 1:**
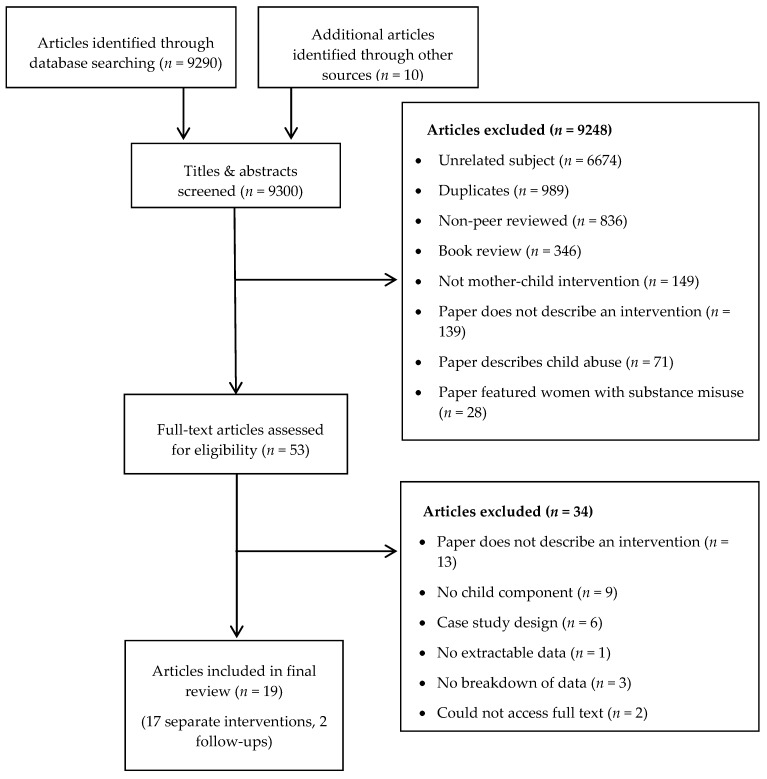
Flow diagram for article selection.

The combined electronic and hand searches produced a total of 9300 results. No authors responded with additional/unpublished work. After eliminating duplicates, book reviews, non-peer reviewed, unrelated articles, and other articles that did not meet search criteria, 53 texts were screened in full. From these articles, a total of 34 were deemed ineligible, and 19 (17 separate interventions, 2 follow-ups) were subsequently entered into the review. Figure 1 depicts the full screening process and outlines motives for exclusion. The findings of this review are presented according to the following categories: (1) separate interventions; (2) joint interventions; and (3) combined interventions; with the structure and characteristics of each described, followed by the results. See Table 1 for all included studies.

### 3.2. Overview of Featured Interventions

The included interventions span 24 years, from 1992–2016, and have a total sample size at intervention entry of *n* = 2413 (complete figures for rates of drop out are not available). This review identified 10 RCTs and seven non-randomized interventions from four different countries (USA: 14, Canada: 1, Wales: 1, Israel: 1). Participants were recruited from IPV/family homeless shelters (*n* = 9), family programs for domestic violence (*n* = 2), multiple community locations, including health clinics, education centers, shelters and outreach services (*n* = 5), and clinical facilities (*n* = 1). Most commonly, interventions implemented treatment programs for children aged between three and twelve. In total, 1175 mothers and 1256 children participated in the interventions, with an average sample size of 64 and 63 respectively. On average, the 17 interventions highlighted in this review scored 3 according to GRADE criteria (see Table 1 for full overview).

#### 3.2.1. Separate Interventions

We use the term *separate* to account for psychosocial interventions taking place simultaneously for mothers and children, but independently from one another. Often these were held at the same time and at the same location. Four of these interventions [38,39,40,41] implemented 10- or 12-week, broadly psychoeducational programs lasting on average one hour. Sessions for mothers focused on topics including: parenting skills and appropriate praising/reprimanding; positive expression of emotion; enhancing self-esteem and mental well-being; promoting prosocial child behavior; safety planning; setting goals for the future; and how to create and maintain successful interactions. For children, where sufficient numbers existed, children were grouped into similar ages and the structure of sessions was tailored accordingly. Topics loosely followed those of mothers and included: mastery of behavior; managing feelings; dealing with conflict between peers; recognizing violent behavior in others; keeping safe; and taking responsibility for their own behavior. These interventions did not comprise an element of mothers and children in the same session.

#### 3.2.2. Joint Interventions

Four interventions treated mothers and children in a joint intervention. Mother and child attended these interventions together and did not receive psychosocial support independently of each other. The first, by Waldman-Levi and Weintraub [42] involved 8 sessions of 30 min. This ‘family intervention for improving occupational performance’ (FI-OP) addressed difficulties in mother-child interactions and deficits in children’s play functioning. Similarly, Smith and Landreth [43] tested the effectiveness of filial therapy with mothers and children in 12 sessions over 3 weeks. Filial therapy also uses child-centered, play-oriented principles in order to strengthen the relationship. Jouriles et al. [20,44] implemented a joint intervention taking place in participant’s homes (different from the rest of studies that took place in clinical or research facilities), with a specific focus on conduct problems in children. Their project *Support* taught mothers to encourage and facilitate appropriate behavior in the mother-child relationship. It also had components of emotional support, problem solving and effective communication, all with the aim of reducing conduct problems. Project Support was embedded in principles of child management skills and parenting, as well as instrumental and emotional support aimed to reduce mother’ psychiatric symptomatology long-term [45].

#### 3.2.3. Combined Interventions

Nine interventions supplemented separate intervention programs for mothers and children with joint sessions which they attended together, the structure of which varied greatly. Smith [46] (see also McManus and colleagues [47] for full program details) tested a 10-week psychoeducational program with sessions lasting up to 2.5 h. The first half of sessions were spent with mother and child together, working jointly on activities, which aimed to help share their experiences of the abuse and to acknowledge their related feelings and concerns while supporting one another. The second half of sessions was in separate groups, whereby a structured program (Domestic Abuse, Recovering Together: DART) was implemented. In another interventions, McWhirter [48] combined separate mother and child work with a joint family session. Two themes were employed across both treatment elements: goal-oriented and emotion-focused. The goal-oriented women’s group drew on cognitive behavioral and motivational interviewing techniques, whereby women focused on decreasing non-adaptive coping strategies and increasing adaptive ones. Goals were either relational (e.g., fostering healthy bonds with their children), personal (e.g., increasing awareness for personal feelings), or functional (e.g., drink alcohol less frequently). The emotion-focused group comprised behavioral and therapeutic components. Each session included a psychoeducational element that presented information, which was then assimilated via a gestalt approach that encouraged women to process information, thoughts and feelings in a way that allowed for behavioral changes. Separate sessions of similar themes were implemented for children in parallel. This was followed by a family-based activity and discussion, where they were presented a summary of the sessions.

Other interventions, both RCTs, that implemented a combination of separate and joint interventions include Cohen et al. [49] who tested trauma-focused cognitive behavioral therapy with a concentration on psychoeducation about trauma; developing individualized relaxation skills to manage stress, expressing and modulating upsetting feelings, and cognitive coping skills. Within the joint child-parent sessions that followed, the child was encouraged to share IPV experiences directly with the mother. Lieberman and colleagues [17] implemented a child-parent psychotherapy program, with the aim to facilitate child–parent interactions. This was guided by the child’s free play with developmentally appropriate toys selected to elicit unprocessed distress and foster social interaction. The initial assessment included individual sessions with the mother to agree on the course of treatment, and to plan how to explain the treatment to the child. CPP aims to target maladaptive behaviors, supports developmentally appropriate interactions, and guides the child and the mother to create a joint narrative of the traumatic events. Weekly joint sessions were interspersed with individual work with the mother as clinically indicated. Sullivan et al. [50] ran simultaneous groups for mothers and children where they addressed safety planning, trauma-related issues and other psychosocial effects in the aftermath of violence, self-blame and conflict resolution skills. This was followed by a multi-family group that aimed to facilitate communication and sharing of the experiences gained during the simultaneous groups.

Graham-Bermann and colleagues compared a range of intervention types within their series of RCTs. The team began by implementing a joint 10-week psychoeducational program for mothers and their children [13], which led to the development of the Mom’s Empowerment Program (MEP) and the Pre-Kids’ Club (PKC) [15,27] that could be independently run. The MEP aims to empower women through discussing the impact of the violence on themselves and on their children; to optimize parenting skills; to provide a safe place to discuss parenting fears and worries; and to build connections for the women in the context of a supportive group, thereby reducing symptoms of depression and enhancing their parenting skills. The child component (PKC) targets their knowledge and beliefs about IPV, their emotional adjustment, and their social behavior.

Carter et al. [51] were the only authors identified in this review to combine group treatment and family therapy, that was preceded by separate sessions. This multi-level approach allowed for children and mothers to learn and speak about their IPV experiences and provide a healing environment. Critical issues arising in separate group sessions were followed up within family therapy.

### 3.3. Outcomes of Interventions

All 17 interventions conducted pre- and post-assessments, eight of which combined these with follow-up assessments ranging from 3 months [38] to 2 years [45]. A total of 34 different outcome measures were used in these interventions (16 for mothers, 18 for children).

#### 3.3.1. Separate Interventions

Child adjustment (using the Child Behavior Checklist, CBCL; [53]) was assessed in three interventions; two of which observed promising reductions overall, particularly in internalizing behaviors in children from pre- to post-treatment [39,41]. In addition, female children in the study by Macmillan and Harpur [41] were observed to achieve a greater decrease in internalizing behaviors at eight months’ follow-up. Children in the these programs were additionally found to have improved domestic violence related skills [39] (e.g., able to identify the impact of the abuse on oneself, demonstrating knowledge of domestic violence and power/control dynamics, able to differentiate between appropriate and inappropriate expressions of anger), and improvements in their knowledge of abuse and safety planning [41]. Mothers were observed to have lower parenting stress [41] and improved parenting skills [39], but no significant reductions in symptoms of depression, anxiety or trauma [38].

#### 3.3.2. Joint Interventions

For joint interventions, child behavioral problems were addressed in two separate programs and found that conduct problems were brought to within normal range [20] and only two out of 13 children met clinical threshold after two years [45]. Similarly, despite reductions in conduct problems for both treatment and comparison groups, more rapid decreases were observed for project ‘Support’ children, although by the fifth assessment (16 months post assessment) and at 2 years follow-up there was no difference between groups. Mothers in these interventions were reported to have diminished distress and improved child management skills, with improvements still below clinical range at 24 months’ follow-up, improved psychiatric symptoms and less harsh parenting.

With regard to the two play-oriented programs, Smith and Landreth [43] demonstrated that children in filial therapy–as facilitated by their mother–improved as much as the comparison groups that were facilitated by professional therapists. This type of treatment was also more adept at reducing externalizing and internalizing behaviors, and aggression than the comparators. It also allowed mothers to more effectively convey empathy to their children, as well as communicating their acceptance of their child and leading them to be self-directive. Waldman- Levi & Weintraub [42] observed a significant improvement in the family group for sensitivity and limit setting, and for children’s play skills. However, in terms of interaction, there was no difference in playfulness, involvement, sensitivity or reciprocity.

#### 3.3.3. Combined Interventions

Trauma symptomatology were reportedly reduced to within normal range in children, in four combined interventions that measured this outcome [13,17,49,50] including lower levels of avoidance, hyperarousal and anxiety. Sustained improvement in trauma symptomatology was also recorded in interventions by Graham-Bermann and Miller [15] and Lieberman et al. [52] at eight and six months’ follow-up, respectively. Lieberman and colleagues [17] additionally found a significant reduction in avoidant symptomatology for mothers in their study.

Child adjustment was assessed in four combined interventions [13,17,49,50], with treatment conditions proving more effective in reducing child externalizing and internalizing behaviors than controls. Graham-Bermann et al. [13] also observed significantly greater reductions in the joint sessions post-treatment to follow-up when compared to children-only groups. Additional findings for children in combined interventions include increased self-esteem and emotional well-being [48], and significant drops in intrapersonal distress, somatic symptoms, interpersonal relations and behavioral dysfunction [51]. With regard to child attitudes, three interventions found positive results: improved beliefs regarding violence [13], fewer worries about their mothers being vulnerable to violent injury [51], and reducing their own self-blame for violence in the home [50]. Carter and colleagues [51] found there to be no significant changes to social skills for children following treatment.

Mothers in the combined interventions were recorded to have feelings of greater social support (emotion-focused group), reduced family conflict (goal-oriented group), and fewer symptoms of depression and increased self-efficacy for women in both groups [48]. In addition, reduced isolation and life stress was observed by Sullivan et al. [50], as well as significantly greater self-esteem, greater confidence in parenting abilities and more control over their child’s behavior [46] and improvement in depression symptoms post-treatment [40].

## 4. Discussion

This review sought to map interventions for women who are directly affected by IPV, and their children who are secondary victims, and to build on similar existing work that address the needs of those affected by violence in the home. In total, 17 separate interventions met select search criteria, of which 4 were held separately for mothers and children, four were conducted only in joint sessions, and 9 were a combination of both. This review serves as a useful starting point for efforts to further the development of interventions for IPV-exposed children, and children who are secondary victims of trauma. Unlike other reviews, we aimed to delve deeper into the underlying mechanisms by which mothers directly affected by IPV and children who are exposed to it, can navigate their pathway toward recovery. In particular, Rizo et al. [30] reviewed 31 articles featuring programs that either directly or indirectly target IPV-exposed children. Owing to the complex interaction patterns by which it is believed children are impacted by exposure to IPV in the home, the present article built on their work by considering programs with both child and caregiver components. We reviewed nine of the same articles as these authors. In addition, Austin et al. [33] reviewed 19 interventions for women parenting in the context of violence, but provided no explanation as to why this might occur. We reviewed 12 of the same articles as these authors.

Main findings of this review highlight interventions held separately for mothers and children as being successful in targeting adjustment behaviors and parenting stress, and at enhancing IPV related coping skills. Interventions that worked with mother and child in a joint session were particularly useful in regard to child-centered, play-oriented principles as well as improving conduct problems. Studies implementing a combination of separate and joint working were seemingly more successful in improving a wider range of outcomes, including traumatic stress, child adjustment, self-esteem, social problems and positive attitudes. as well as increasing social support, self-efficacy, depression and confidence for mothers. These aspects address the problems identified for women exposed to IPV, in existing research. In addition, implementing elements of play within joint treatment has been observed to be successful in improving child adjustment and mother-child interaction, and a series of joint home-based interventions have proven useful in targeting conduct problems in children. The results of this review highlight contrasting mechanisms by which an improvement in IPV-related difficulties is sought, and there are distinct characteristics that appear to be key in successful interventions for mothers and children. These potentially occur (1) individually or (2) collaboratively, and to further understand the mechanisms by this may occur, one possible theory is outlined below.

### 4.1. Theory of Change

The first is an individualistic mechanism by which change may occur observed in separate interventions and is a response to violence by managing one’s own behavior. By coping with the personal experience–whether as a mother who is a victim of direct violence, or a child who is exposed to it in the home–the most salient or observable problems have the opportunity to be explored in separate sessions. In line with previous research, the child’s own poor outcomes resulting from exposure to violence in the home, could be addressed using an individualistic mechanism. In this review, individualistic interventions most often feature a psychoeducational framework, and appear to be successful in addressing the psychosocial problems that arise as a result of exposure to violence in the home; managing child adjustment and adverse coping skills, as well as providing social support within the group environment. In separate sessions, children are able to learn and understand, age-appropriately, about the violence that occurred within their families, talk openly about their experiences, and engage in activities that promote processing and recovery. Children who show resilience would benefit from separate sessions, as they are likely to be more confident to share their experiences [54]. Separate sessions that take place in parallel also enable women to attend without concern about child care [55], and can prevent a mother becoming flooded with intrusive memories, and failing to modulate her affect towards the child [17]. However, these types of interventions that take place simultaneously, largely fail to conceptualize children as agentic subjects during treatment [56], capable of maneuvering the process towards joint recovery, and they do not allow the crucial mother-child bond to be strengthened within a therapeutic environment.

The second is a collaborative mechanism of change with a focus on combining the benefits of mothers and children working both together and independently. As well as managing one’s own behavior, as observed in individualistic interventions outlined above, interventions with a collaborative mechanism seek to go beyond this, tapping into a ‘bilateral’ model [56] whereby both are active participants. Given that parents naturally move with time to view their child as capable of having their own needs and desires [57], the notion of allowing children to be active participants in the process of recovery relating to IPV could be particularly useful. This is not to say that the primary aim of children living with IPV is to support their mother, however, working together in joint interventions creates opportunities to harness a child’s agency, which is said to be a key factor in moderating the dyadic outcome [17]. Using the tools to manage one’s own behavior gained in separate interventions (e.g., processing trauma, improved communication and problem-solving skills), mothers and children can share IPV experiences, learn to express and modulate upsetting feelings and plan how to proceed with treatment all within a healing environment.

Additionally, as summarized in this article, a mother’s own stressful experience caused by IPV, can manifest in her parenting, and thus reflect in the child’s own functioning. Collaborative mechanisms can address this transaction. By being active parties in the experience of IPV, a deeper, more substantive change has the potential to take place; the mother demonstrates her availability to the child and with a primary focus on their bond, once strengthened, this can lead to improvements in other areas. Positive reciprocal relationships are beneficial to both parties in promoting recovery following IPV in the home [56]. Therefore, it appears that combined interventions are incredibly valuable. The collective autonomy and agency of children are believed to be protective factors that can serve to facilitate adjustment and interaction, as well as the potential repercussions of witnessing traumatic events. As part of the DART program for mothers who have experienced IPV and their children who are exposed to it in the home, Smith [24] describes a theory of change that is essentially a framework to guide providers of care through a pathway towards recovery (see pages 14, 52–53 for more detail). The first three stages outline common circumstances surrounding IPV, and the impact that this has on individual functioning and reciprocal relationships. The next stage accounts for an intervention such as DART to take place, and the following are outcomes that contribute towards recovery. In light of the results outlined in this article, it has the potential to be adapted and tested to include the impact of individual and collaborative change mechanisms as an intermediary stage in the pathway.

Nonetheless, despite the potential benefits of combining individualistic and collaborative interventions, at a pragmatic level shelters for families affected by IPV may not have the facilities to continuously provide such structured, resource-oriented modalities. For example, CPP [17] requires commitment of one year to the treatment program, which may not be attainable for some mother-child dyads who face instability across many life aspects. Holt et al. [26] suggest that once primary needs have been met, support should fall along a continuum; combining short and long-term, individual and group-based programs, through existing networks of family and community support.

### 4.2. Limitations of This Review

Despite the range of promising findings outlined here, generalizability of these results should be approached with caution for a number of reasons; arising from both the included studies and regarding the review methodology itself.

As documented, more than 30 assessment tools were used to measure outcomes in these programs, and not all interventions in each category (separate, joint, combined) tested the same concepts. What this could mean is that teasing apart the mechanisms by which change occurred within separate or joint interventions, is problematic. In addition, if there are numerous tools of varying reliability being used to measure many of the same constructs, there is a question as to whether interventions can authentically be compared. This may be a reflection of the myriad psychological effects observed in mothers and children exposed to IPV, however it suggests a clear lack of consensus regarding the aims of treatment, the clinical effectiveness of outcome measures, and an overriding disagreement on how to support mothers and children impacted by IPV. It also leads to scattered knowledge on the subject with regard to research implications. With these limitations in mind, future research would ideally be conducted to add weight to the findings.

There is also a need for the replication and follow-up of the interventions included in this review with diverse groups, in order to be able to extrapolate the findings. Despite the extensive evidence from Garcia-Moreno et al. [3] that violence affects many communities worldwide, only three interventions were conducted outside of the USA [35,37,41]. Access to and quality of treatment can differ tremendously, and research conducted in North America is not wholly applicable to all populations. Moreover, the studies included in this review originated from a small number of research teams (8 of the 19 articles were from 2 separate research groups). Replication by other parties would again add weight to the success of these interventions.

Methodologically, randomized controlled trials were not universally employed in these interventions, and many of those who did, failed to adequately describe randomization procedures [51] and other methodological minutiae. This could relate to the date in which the standardized reporting guidelines, Consort [58], were published, but it meant that RCTs had the potential to be downgraded from a four to a three using GRADE criteria. Furthermore, sample sizes were often less than 100, there was frequently no follow-up and there was limited information given as to whether mothers and children were still experiencing IPV. This is particularly important given that interventions may have less success if IPV is still occurring at home. Attrition was a problem across the board, which to a certain extent is expected among this client group, who face enormous instability and uncertainty [50]. However, approximately only 2% of children exposed to IPV live in shelters [33], which suggests that recruitment to interventions ought to include wider local outreach to identify those in need elsewhere. In addition, few interventions implemented multiple age groups within their programs for children, with even fewer extending up to 18 years. Thus, interpreting age-specific changes and outcomes is not possible.

In terms of the review process, the exclusion of children as direct victims of violence in the home is a potential limitation of this review that would ideally be addressed in future research and programming (equally discerning whether there is a lifetime experience of direct violence for children that has since stopped). In reality, distinguishing children on this factor is arbitrary as both direct and indirect victimization are likely to co-occur. However, much of the current literature on the effects on children do not control for the variable of child abuse, which makes it difficult to separate the effects [31]. Furthermore, the exclusion of mothers with substance misuse problems could have led to a selection bias in this review whereby a cohort of women were not captured. Substance misuse may play a role in many families who have experienced IPV, and even be a factor that impacts parenting thereafter. Future research would ideally account for this.

With both methodological strengths and limitations in mind, these results bring together varied treatment elements, a range of assessment measures and varying degrees of success in improving outcomes for mothers and children impacted by IPV in the home. A lack of raw data prevented a full meta-analysis from being conducted, but it could be inferred tentatively that combining separate and joint mother-child interventions have a wider applicability to the outcome of IPV in the home on mothers and children.

### 4.3. Future Research and Practice

On the basis of this review, there are many potential avenues for future research to extend and consolidate the knowledge and understanding on issues of IPV in the home, and a child’s role in this. Primarily, there needs to be a wider replication of interventions in terms of mother-child relationships, child functioning and maternal mental health. This includes with minority groups, in diverse populations, repeated more than once and with increasingly larger samples. Only then can these interventions be deemed truly successful. In particular, interventions that address the agency of both mother and child in collaborative interventions could add to our understanding of the reciprocity of mother and child recovery after IPV. In addition, we would welcome studies that strengthen effective implementation of stepped-care programs. Further, both clinical and research-oriented work might look to reframe the terminology by which the issue of IPV against women who are parents, is addressed. While women who experience IPV from their spouses are not necessarily to blame, ‘violence by men’ is a way to shift the focus from mother’s ‘problematic’ parenting to the root cause of the problem, which is the violence perpetrated within relationships [9]

Children are often equal victims in experiencing neglect, or indeed physical, emotional or sexual abuse from their parent perpetrator. They often become victims to disproportionately high levels of maltreatment. This fine margin where children move from secondary victims, to experiencing violence directly is important for treatment and could be elaborated on further in future research. Witnessing violence can also make children more prone to their own use of violence [43], and this notion should not be overlooked in treatment. IPV is specifically directed at the child’s parent by a perpetrator with whom the child almost always has an ongoing emotional relationship, albeit often painful or conflictual. Enhancing a child’s agency in such circumstances could be an effective tool to reduce the likelihood of engaging in their own violent behavior. Ideally, a holistic treatment encompassing methodological elements and mechanisms of change highlighted in this review, would serve to address the individual needs of mothers and children.

Moreover, positive reciprocal interactions may have been lost within the dynamics of violence among families, but ought to be overtly addressed in treatment of any kind. Collaborative modalities appear to be a way for mothers to understand and reflect on the child’s perspective. For many children, difficulties verbalizing feelings and thoughts can be experienced, particularly if these are related to experiences such as IPV [59]. For younger children, incorporating play in treatment would not only increase the child’s agentic state, but provide the setting for parents to improve certain behaviors, to enhance the interaction. In addition, trauma-oriented work with this population appears to be particularly successful for learning about the circumstances at home and preparing for real or perceived dangers, which likely underpins the hyper-vigilance aspect of posttraumatic stress disorder for this group.

In addition, considering the time for some women to be permanently separated from their abuser and the frequency with which some women return to their relationships [10,27,39], it is worth reiterating that a distinction between mothers who have left their violent homes, and those still in contact with an abusive spouse or partner is challenging. Women (and children) may still be affected by violence, or coercive or controlling behavior, and these may even impact the success of programs.

## 5. Conclusions

Despite a vast evidence-base on the implications of IPV on families, little consensus regarding treatment and support appears throughout the literature, or indeed clinical practice. One explanation of this is the wide range of interventions that have been implemented to treat mothers and children. This review identified 17 studies published up to 2016 and highlighted two distinct mechanisms by which the needs of mothers and children are targeted. This article adds to the growing evidence base, highlights the importance of resources that are needed in the community, and brings further awareness to the concern of violence in the home. It also confirms the positive impact on well-being that programs for IPV victims can have. The evidence-base overall could benefit from testing and replicating a combination of the results found in this review.

## Figures and Tables

**Table 1:** Overview of included interventions.

Author, Year, {Reference]	Intervention Recruitment Locations	Study Design	Age of Children	Sample Size	Study Aims	Brief Intervention Description	Control Group	Assessment Time Points	Outcome Measures	Findings	GRADE Result
**Separate interventions**											
Basu et al. 2009 [38]	USA Community	Randomized group intervention	3–12	Children: 20 Mothers: 36	To implement a community-based, manualized, psychoeducational intervention program targeting mothers and children exposed to IPV	10 weeks, 1.5 h/week for mothers, 1 h/week for children. Psychoeducational: Focus on different theme relating to IPV	Waitlist	Pre- & 3 + 6-month FU	Mothers: severity of violence, distress, depression, trauma Children: perceived competence & social acceptance, feelings toward IPV at home	No significant differences for depression, anxiety or trauma symptoms across the groups. The intervention group had the lowest levels of depression and anxiety symptoms compared to both the intervention and early termination groups over time. Children in the CG showed a decrease in anxiety and depression symptoms relative to the other groups immediately post intervention but not in the 3- or 6-month assessments. There were no significant differences for trauma symptoms.	3
Becker at al., 2008 [39]	USA Community	Non-randomized intervention	3–17	Children: 106 Mothers: 56	To implement a culturally influenced intervention program involving a sample largely identifying as from Asian and Pacific Island descent.	12 weeks, 1.5 h/week. Focus on different theme relating to IPV	-	Pre- & post-intervention	Mothers: IPV related skills, parenting practices Children: IPV related skills, behavior checklist	Children had significant improvement in ratings of violence-related from pre-post treatment. Significant decrease in internalizing and externalizing scores. Significant decrease in the proportion of children with clinically significant posttreatment psychopathology. Parents were observed to have significant improvement in their IPV related skills and parenting practices.	3
Graham-Bermann, et al., 2015 [40]	USA IPV shelters	RCT	4–6	Mothers & children: 120	To compare the adjustment of children exposed to severe IPV who participated in the Pre Kids’ Club (PKC) while their mothers participated in the Mom’s Empowerment Program (MEP)	5 weeks, 2 sessions/week. Mothers: to enhance social and emotional adjustment. Children: each session focuses on different topics related to IPV	Waitlist	Pre- & post- intervention 8 months FU	Mothers: severity of violence. Children: behavior checklist (internalizing only)	There was no statistically significant decrease in internalizing problems over time in the control group. For female children in the treatment group, there was a statistically significant decrease in internalizing problems at the 8-month follow up point. Under a per-protocol specification, there were statistically significant differences between the treatment and comparison groups.	3
Macmillan & Harpur, 2003 [41]	Canada Community	Non-randomized intervention	6–12	Children: 47 Mothers: 39	To describe the well-being and functioning of this sample of children and parents who are living in the community and seeking out treatment.	10 weeks, 1.5 h/week. Children: addressing posttraumatic stress issues, IPV related skills, relaxation. Mothers: promoting relationship building, positive discipline practices.	-	Pre- & post- intervention	Parents: parenting stress Children: behavior checklist, depression, anxiety, trauma-related sequelae, understanding of abuse	Children’s behavior problems (externalizing, internalizing, and total score) were significantly lowered, while children’s scores on the knowledge forms were significantly increased. Parenting stress significantly lowered.	3
**Joint interventions**											
Jouriles et al., 2001 [20]	USA IPV shelters	RCT	4–9	Mothers & children: 36	An experimental evaluation of a programme designed to reduce conduct problems of children of domestic violence victim mothers.	8 months, 1.5 h/week. Child management skills	Monthly telephone calls	Pre-intervention 4, 8, 12, 16 months FU	Mothers: severity of violence Children: behavior checklist Mothers & children: child management	Significant improvement in externalizing over time. Slightly higher mean level of child management skills at assessment 3, and improving more rapidly in families in treatment condition. Mother’s psychological distress diminished over time. Level of conduct problems in intervention arm brought to within normal range, mothers gained more rapid and greater improvements in child management skills.	3.5
Waldman Levi & Weintraub, 2015 [42]	Israel IPV shelters	Non-randomized intervention	3–5	Children: 37 Mothers: 37	To examine the efficacy of filial therapy for mothers and their children in IPV shelters.	8 weeks, 30min/week. Opening (5 mins), joint play (20 mins), closure & separation (5 mins).	Free play time	Pre- & post- intervention	Mothers & children: interactive behavior Children: play skills, playfulness.	Children’s play skills significantly improved in the FI-OP group for sensitivity and limit setting. No differences fond in involvement, reciprocity, negative states. Children’s play skills significantly improved, but not regarding material management or participation. No difference in playfulness between groups.	2.5
Smith & Landreth, 2003 [43]	USA IPV shelters	Non-randomized intervention	4–10	Children: 11 Mothers: 11	To determine the effectiveness of intensive filial therapy as a method of intervention with child witnesses of domestic violence.	Filial therapy 2–3 weeks, 12 sessions, 1.5 h/session Combined parent training session and parent-child play session.	Sibling group therapy	Pre- & post intervention	Mothers & children: empathy Children: behavior checklist, self-concept.	Intervention group demonstrated significant improvement on all measures. Children in the intensive individual play therapy group scored significantly higher in self-concept than children in the filial therapy. There were no significant differences between the intensive filial therapy experimental group and the intensive sibling group play therapy comparison group on self-concept scores. Mothers achieved significantly higher levels of positive behavior.	3
Jouriles et al., 2009 [44]	USA IPV shelters	RCT	4–9	Mothers & children: 66	To replicate and extend findings from initial findings (Jouriles et al. 2001).	12 months, weekly home visits. Child management skills.	Month-ly phone calls	Pre-intervention 4, 8, 12, 16, & 20 months FU	Mothers: severity of violence, parenting, psychological aggression, psychiatric symptoms, traumatic symptoms. Children: conduct problems, frequency of behaviors, oppositional behavior.	Child conduct problems decreased more rapidly in the intervention group. For the follow-up period, conduct problems continued to decrease in the intervention group, but not in the comparison group. Although oppositional child behavior decreased more slowly than the other measures of child conduct problems, child behavior still decreased more rapidly in the intervention group than the comparison group during both the intervention and follow-up periods. During the intervention period, inconsistent and harsh parenting behaviors decreased in the Project Support group, and in the comparison group, with more rapid decreases in the Project Support group. During the follow-up period, no changes in inconsistent and harsh parenting behaviors emerged in either of the groups. Maternal psychiatric symptoms decreased during the intervention period in the Project Support group, and in the comparison group.	4
Macdonald et al., 2006 [45]		Follow-up		Mothers & children: 36	To assess the effects of Project Support on children’s conduct problems 24 months following the termination of services (32 months following shelter departure).			24 months	Mothers: aggression, contact w/partner, recurrence of violence. Children: oppositional behavior, behavior checklist, internalizing problems.	Only 31% of children still in clinical level of conduct problems at either 16 months/32-month assessment points (compared to 71% in comparison group). Externalizing scale scores for intervention and comparison conditions at the 24-month follow-up assessment did not differ significantly from one another. Mean levels of internalizing problems did not differ between the treatment and comparison groups at the 24-month follow-up assessment. However, there were differences in the proportion of children in each group exhibiting clinical levels of internalizing problems.	3.5
**Combined interventions**											
Graham-Bermann et al., 2007 [13]	USA IPV shelters	RCT	6–12	Mothers & children: 181	To promote alternatives to aggression and address children’s beliefs about violence	10 weeks, Mothers: building parenting competence Children: understanding IPV-related behavior	Waitlist	Pre- & post-intervention 8-month FU	Mothers: severity of violence, social desirability Children: behavior checklist, attitudes about IPV	Individual CM children displayed significantly greater improvement from baseline to post-intervention relative to controls in externalizing behavior problems and attitudes in the two-level model comparing change in individuals assigned to different conditions. Individual children in the CM condition made significantly greater changes in externalizing behavior problems from posttreatment to follow-up when compared with children in the CO condition. Significant deterioration in attitudes for individual CO children, suggesting that mothers may influence their children’s beliefs and attitudes about violence after participating in the intervention programme themselves	4
Graham-Bermann & Miller, 2013 [15]	USA IPV shelters	RCT	6–12	Mothers & children: 181	To assess the efficacy of a group intervention in relieving traumatic stress symptoms for women exposed to IPV.	10 weeks Mothers: building parenting competence Children: process feelings re: IPV, IPV related skills.	Waitlist	Pre- & post- intervention 8-month FU	Mothers: severity of violence, PTSD, social desirability Children: -	The more social desirability the less reported trauma symptoms. Significant reduction in traumatic stress symptoms for all 3 conditions from baseline to end of treatment. From baseline to follow up was bigger change.	3
Lieberman et al., 2005 [17]	USA Mixed clinical locations	RCT	3–5	Children: 75 Mothers: 75	To evaluate the efficacy of child-parent psychotherapy (CPP) compared with case management plus separate treatment.	CPP 50 weeks, 60min/week. Children: free play Mothers: managing the child and their experiences of IPV Weekly joint sessions to enhance interactions	Case manage-ment & usual care	Pre-intervention, 6 months into treatment	Children: exposure to community violence: behavior checklist, trauma. Mothers: life stress, psychiatric symptoms, traumatic stress disorder.	Intervention group had a significant reduction in the number of trauma symptoms, whereas the comparison group did not. Significant reduction in behavior problems. Significant reductions in maternal avoidant symptoms for the intervention group post-intervention. Decline in PTSD diagnosis for mothers in both groups, although not statistically significant.	4
Smith, 2016 [46]	Wales	Non-randomized intervention	7–11	Children: 147 Mothers: 147	To enhance the mother–child relationship, in addition to supporting other aspects of their recovery.	10 weeks, 2.5 hrs. Increase mother’s confidence in parenting.	-	Pre- & post-intervention	Mothers: self-esteem, locus of control Children: self-esteem, well-being Mothers & children: acceptance and rejection	Mothers had significantly greater self-esteem, more confidence in their parenting abilities and more control over their child’s behavior. They were also more affectionate to their child. Children experienced fewer emotional and behavioral difficulties following DART. Children appeared to be experiencing significantly fewer emotional and behavioral difficulties following DART. The children’s self-esteem scores improved but this was not statistically significant.	3
McWhirter, 2011 [48]	USA Family homeless shelter	Randomised group intervention	6–12	Children: 48 Mothers: 46	To assess the clinical effectiveness of emotion-focused and goal-oriented treatments to reduce IPV and increase psychosocial well-being of women and children previously exposed to IPV	5 weeks, 1 hr/week mothers; 45min/week separate child sessions + 60min mother-child group session. Assigned to either emotion-focused or goal-oriented for both parts.	Active control	Pre- & post- intervention	Mothers: family conflict, family bonding, quality of social support, depression, self-efficacy, readiness to change, alcohol use. Children: general psychological well-being, peer conflict, family conflict, self-esteem.	Children in both groups reported decreases in family and peer conflict and increases in state of emotional well-being and self-esteem. Women in both groups reported decreases in depression and increases in family bonding and self-efficacy. Significantly greater decreases in family conflict were reported among goal-oriented participants and significantly greater increases in social support were reported among emotion-focused participants.	4
Cohen et al., 2011 [49]	USA Community IPV center	RCT	7–14	Children: 124 Mothers: 124	To test whether abbreviated TF-CBT would improve children’s total IPV-related symptoms significantly more than usual care: child-centered therapy (CCT).	Individual TF-CBT. 8 weeks, 45mins/week. Developing positive coping strategies in separate sessions. 2 joint sessions to share IPV experiences.	Usual care	Pre- & post-intervention	Children: trauma, anxiety, depression, behavior checklist, cognitive functioning, verbal & non-verbal intelligence.	The intervention group experienced significantly greater improvements in overall trauma score, hyperarousal, and anxiety.	4
Sullivan et al., 2004 [50]	USA Community	Non-randomized intervention	8–16	Children: 79 Mothers: 46	To address the needs of parents and children regarding coping abilities, parenting skills, safety planning skills, and the effects of post violence stress	9 weeks. Focus on different theme relating to IPV	-	Pre- & post- intervention	Mothers: parenting stress Children: behavior checklist, trauma symptoms, anxiety, depression, anger, dissociation, self-blame.	For child behavior checklist, only 3 of the 14 measures were significantly reduced from pre-test to post-test: anxious or depressive behaviors, internalizing behaviors, and externalizing behaviors. Findings suggest the intervention programme significantly reduced trauma symptoms in the clinical subsample and significantly reduced the Anger subscale in the entire sample. Within the parenting stress scale child domain: adaptability, mood, reinforcing parent, and distractibility or hyperactivity were significant. In the parent domain, isolation, life stress, and health were significantly improved at post-test. However, the findings on the latter two subscales may lack clinical significance because both the pre-test and post-test health scores were in the non-clinical range and both the pre-test and post-test life stress scores continued to score in the clinical range. Children’s self-blame was significantly reduced at post-test in the overall sample.	2
Carter et al., 2003 [51]	USA IPV Violence programme	Non-randomized intervention	4–18	Children: 192 Parents: 64	To build safety planning skills, self-esteem, ways of expressing feelings, prosocial skills, conflict resolution skills, parent-child relationship skills, identify and strengthen support systems; and provide an atmosphere for self-disclosure and therapeutic interventions to heal trauma responses.	12 weeks, 1.5 h/week. Individual, group, family therapy services. Focus on different theme relating to IPV	-	Pre- & post-intervention	Parents: parenting stress. Children: occurrence of behavior change, ability to express emotions, social skills & adaptive functioning, PTSD, self-concept, family worries, family stereotypes.	Statistically significant decrease in intrapersonal distress, somatic symptoms, interpersonal relations, social problems and behavioral dysfunction, although interpersonal distress and interpersonal relations remained clinically significant. There were no significant changes in social skills following treatment. However, parents reported significantly fewer behavior problems in their children following treatment. Following treatment, children reported having significantly fewer worries about their moms and themselves being vulnerable to injury.	3
Lieberman et al., 2006 [52]		Follow-up		Children: 50 Mothers: 50		Monthly telephone calls		6-month FU	Mothers: psychiatric symptoms Children: behavior problems.	Intervention group had significant reductions in child behavior problems and maternal symptoms. Decline in symptom severity was statistically significant only for the CPP group mothers.	

CCT: child-centered therapy, CG: control group, CO: child only. CM: child plus mother intervention, CPP: child-parent psychotherapy, DART: Domestic Abuse Recovering Together, FI-OP: Family Intervention for improving Occupational Performance, FU: follow-up, IPV: intimate partner violence, MEP: Moms’ Empowerment Program, PKC: pre-kids’ group, PTSD: posttraumatic stress disorder, RCT: randomized controlled trial, TF-CBT: trauma-focused cognitive behavioral therapy, USA: United Stated of America.

## Appendix A

**Table A1:** Search terms ^§^.

Group 1	Group 2	Group 3
famil *	intimate partner violence	Intervention
parent *	domestic violence	Systemic
mother	partner homicide	therap *
father	abuse	Group
caregiver	domestic abuse	individual
guardian	sexual abuse	mediation
family relation *	emotional abuse	psych * intervention
mother-child dyad	physical abuse	prevention
	rape	
	batter *	

^§^ Adjusted according to each database; Literature search completed 30 June 2017; * Word truncation to broaden search to include various word endings or spellings.

*Study eligibility criteria*

*Inclusion:*
All languages
All study types
All sample recruitment locations
All treatment modalities

*Exclusion:*
Violence against males
Father-child interventions
Children born through prostitution
Women with substance misuse issues

*Search methods for identification of studies*

*Databases:*
Medline/PubMED
PsycINFO
Cochrane database
PILOTS

*Hand searches of journals:*
Violence Against Women
Family & Intimate Partner Violence Quarterly
Journal of Family Violence
Journal of Interpersonal Violence
Partner Abuse
Trauma, Violence & Abuse
Anger, Aggression & Violence

*Grey Literature*

Peer reviews; contacting authors in the field; general Internet searches (e.g., Google Scholar, www.opengrey.eu), searching reference lists of key papers, and historical, sociological and human rights literature.

*Main extraction criteria*

Author
Year
Journal
Study location
Study design
Study population
Sample size
Age of mothers/children
Aims
Methods/Intervention outline
Assessment time points
Outcome Measures
Analyses
Results
GRADE
